# Unexpectedly Long Lifetime of the Excited State of Benzothiadiazole Derivative and Its Adducts with Lewis Acids

**DOI:** 10.3390/molecules26072030

**Published:** 2021-04-02

**Authors:** Taisiya S. Sukhikh, Radmir M. Khisamov, Sergey N. Konchenko

**Affiliations:** Nikolaev Institute of Inorganic Chemistry, Siberian Branch of the Russian Academy of Sciences, 630090 Novosibirsk, Russia; khisamov@niic.nsc.ru (R.M.K.); konch@niic.nsc.ru (S.N.K.)

**Keywords:** benzothiadiazole, Lewis acid, photoluminescence, single crystal X-ray diffraction, phosphorescence

## Abstract

We report a study of photoluminescent properties of 4-bromo-7-(3-pyridylamino)-2,1,3-benzothiadiazole (*Py-btd*) and its novel Lewis adducts: (*PyH-btd*)_2_(ZnCl_4_) and [Cu_2_Cl_2_(*Py*-*btd*)_2_{*PPO*}_2_]·2C_7_H_8_ (*PPO* = tetraphenyldiphosphine monoxide), whose crystal structure was determined by X-ray diffraction analysis. *Py*-*btd* exhibits a lifetime of 9 microseconds indicating its phosphorescent nature, which is rare for purely organic compounds. This phenomenon arises from the heavy atom effect: the presence of a bromine atom in *Py-btd* promotes mixing of the singlet and triplet states to allow efficient singlet-to-triplet intersystem crossing. The Lewis adducts also feature a microsecond lifetime while emitting in a higher energy range than free *Py-btd*, which opens up the possibility to color-tune luminescence of benzothiadiazole derivatives.

## 1. Introduction

Chalcogen–nitrogen heterocycles are actively studied owing to the prospects for their application in electronics [1,2] and biomedicine [3]. 2,1,3-benzothiadiazole (*btd*) is a common building block for such applications, featuring electron-withdrawing properties [4,5]. Functionalization of *btd* with donor substituents provides a way to create luminescent compounds, whose emission occurs owing to the push–pull effect [6,7,8]. In addition, *btd* derivatives are prone to secondary bonding interactions such as chalcogen bonding [9] and π–π stacking, which gives rise to the appearance of various photophysical effects: mechanochromism [10], solvatochromism [11], aggregation-induced emission [12], etc.

2,1,3-benzothiadiazole and, in particular, its amine derivatives are able to bind with Lewis acids owing to the donor N atoms of the heterocycle and amino group [13]. Further functionalization of the derivatives with donor groups allows one to expand the set of Lewis base centers [14]. For instance, a number of coordination compounds with *btd* ligands bearing diphenylphosphine [14,15], pyridyl [16,17], triazolyl and pyrazolyl [18] groups were synthesized.

4-bromo-7-(3-pyridylamino)-2,1,3-benzothiadiazole (*Py-btd*, **1**), described recently [19], has a nitrogen atom in the pyridyl substituent, which is known to be a strong Lewis base center. This compound possesses mechanochromic luminescence in solid state, owing to the presence of hydrogen bonds between the amino group and the pyridyl, shortened S···N contacts and “head-to-tail” π–π interactions between benzothiadiazole units. Variation of Lewis acids bound with the pyridyl substituent of *Py-btd* would change the electronic structure of the molecule and secondary bonding interactions pattern in the crystal and, consequently, would result in different luminescent properties of a compound. Particularly, *d*^10^-metal ions, such as Zn(II) and Cu(I), are often used to modulate the luminescence of organic derivatives. They benefit for luminescent applications as earth-abundant nonprecious transition elements. Very recently, a number of Zn and Ag coordination compounds with different 4,7-dipyridyl-2,1,3-benzothiadiazoles was synthesized, and their photoluminescence was studied [20]. Acting as bitopic ligands, these 4,7-dipyridyl derivatives, however, do not comprise a heavy bromine atom. The latter can promote an efficient intersystem crossing in *Py-btd* and its adducts, which ensures their long emission lifetime.

We have synthesized two novel compounds, (*PyH-btd*)_2_(ZnCl_4_) (**2**) and [Cu_2_Cl_2_(*Py-btd*)_2_{*PPO*}_2_]·2C_7_H_8_ (**3∙**2C_7_H_8_) (*PPO* = tetraphenyldiphosphine monoxide) and studied their luminescent properties in comparison with compound **1**. All the compounds exhibit microsecond lifetimes of the excited states, which is rarely observed for organic compounds.

## 2. Materials and Methods

### 2.1. General

Elemental analyses were performed on various MICRO cube instrument for C, H, N and S elements. IR-spectra were recorded on a Fourier IR spectrometer FT-801 (Simex) in KBr pellets. Diffuse-reflectance and photoluminescence spectra were recorded from solid samples on Shimadzu UV-3101 PL and Cary Eclipse spectrometers, respectively. Luminescence decay kinetics were studied on a Cary Eclipse with a Xenon flash lamp (80 Hz, ~2–3 µs pulse width) using an 80-µs delay time and a 50-µs gate time. For diffuse-reflectance spectra, samples were prepared by grinding compounds (0.005 g) with BaSO_4_ (0.200 g), which was used as standard. Diffuse-reflectance spectra were recalculated to absorbance spectra by Kubelka–Munk transformation [21].

### 2.2. Quantum-Chemical Calculations

All calculations were performed in Orca 4.2.1 [22]. Geometry of **1** was optimized by DFT with PBE0 functional [23] and a def2-TZVP(-f) basis set in a gaseous state without any constraints. Dispersion interactions were included by D3(BJ) corrections [24]. Vertical excitation energies were calculated by the TD-DFT method with the same DFT functional but including relativistic effects by the ZORA method and using a ZORA-def2-TZVP(-f) basis set. All calculations were accelerated by the RIJCOSX method [25]. Results of vertical excitation energies calculation were analyzed by TheoDORE 2.2 program [26].

### 2.3. Syntheses

Commercially available anhydrous ZnCl_2_ and CuCl and other chemicals were used without additional purification. Synthesis and purification of 4-bromo-7-(3-pyridylamino)-2,1,3-benzothiadiazole (**1**) and tetraphenydiphosphinooxide (**PPO**) were performed according to earlier described methods [19,27].

(*PyH-btd*)_2_(ZnCl_4_) (**2**)

Amounts of 50.0 mg (0.162 mmol) of **1** and 11.0 mg (0.0811 mmol) of ZnCl_2_ were dissolved in CHCl_3_ (5 mL) by stirring. Yellow precipitate was gradually formed. An amount of 20 μL of concentrated HCl was added and the mixture was stirred for 1 h. Solvent was evaporated under vacuum and precipitate was rinsed with CHCl_3_ and *n*-hexane and dried under vacuum. Yield 39.8 mg (60%). Calculated for C_22_H_18_Br_2_C_l4_N_8_S_2_Zn (825.57): C 32.0, H 2.2, N 13.6, S 7.8. Measured: C 31.6, H 2.1, N 13.2, S 8.0. IR-spectrum (KBr, cm^−1^): 3375 (*w*), 3303 (*w*), 3156 (*w*), 3116 (m), 3072 (m), 2921 (*w*), 1627 (*w*), 1550 (s), 1484 (m), 1388 (m), 1336 (m), 1245 (*w*), 1110 (*w*), 1069 (*w*), 883 (m), 785 (m), 668 (m).

[Cu_2_Cl_2_(*Py*-*btd*)_2_{*PPO*}_2_]·2C_7_H_8_ (**3∙**2C_7_H_8_)

Amounts of 20.0 mg (0.0649 mmol) of **1**, 25.2 mg (0.0652 mmol) *PPO* and 6.4 mg (0.0646 mmol) of CuCl were stirred in toluene (5 mL). The formed precipitate was centrifuged, rinsed with toluene and dried in a vacuum. Yield 38.0 mg (66%). Calc. for C_70_H_56_Br_2_Cl_2_Cu_2_N_8_O_2_P_4_S_2_·2C_7_H_8_ (1771.36): C 57.0, H 4.1, N 6.3, S 3.6. Measured: C 57.2, H 4.0, N 6.1, S 3.6. IR-spectrum (KBr, cm^−1^): 3245 (m), 3056 (m), 2926 (*w*), 2852 (*w*), 1635 (m), 1581 (s), 1550 (s), 1481 (s), 1432 (s), 1383 (s), 1302 (m), 1245 (*w*), 1339 (*w*), 1157 (s), 1094 (s), 1027 (*w*), 883 (*w*), 799 (m), 746 (s), 695 (s), 618 (*w*), 559 (m).

### 2.4. X-ray Diffraction Analyses

Single crystal XRD data for compounds **2** and **3** were collected at 150 K with a Bruker Apex DUO diffractometer equipped with a 4K CCD area detector and a graphite-monochromated sealed tube (Mo Kα radiation, λ = 0.71073 Å, mirror optics). Absorption corrections were applied with the use of the SADABS program [28]. The crystal structures were solved using SHELXT [29] and were refined using SHELXL [30] programs with OLEX2 GUI [31]. Atomic displacement parameters for non-hydrogen atoms were refined anisotropically. Hydrogen atoms were refined in the riding model with the exception of those of the amino group in **2**, which were refined freely with the DFIX restraint on the corresponding N–H bonds. Even relatively large crystals of both **2** and **3**·2C_7_H_8_ gave poor diffraction. Thus, the data were limited to 2 θ of 48.9°. In the case of **3**·2C_7_H_8_, crystals tend towards intergrowth, and the structure was refined as a twin. This resulted in a relatively poor quality of the structure with the final R1 of 7.2%.

CCDC 2065425-2065426 contains the supplementary crystallographic data for this paper. These data can be obtained free of charge from the Cambridge Crystallographic Data Centre via www.ccdc.cam.ac.uk/data_request/cif accessed on 30 March 2021.

Powder XRD was carried at 150 K with a Bruker D8 Venture with a CMOS PHOTON III detector and IµS 3.0 source (CuKα radiation, mirror optics). The samples were prepared by grinding the powders and loading them into 0.5-mm diameter glass capillaries. Diffraction patterns with continuous diffraction arcs were obtained by the φ-scanning method (360°). To improve the powder orientation statistics, 5 scans were taken at different positions of the goniometer along ω from −240° to 0°. Correction for an external standard (Si) and integration were carried out using the Dioptas program [32]. Experimental and simulated XRD patterns were compared using a Topas Academic version 6 software package [33].

## 3. Results and Discussion

### 3.1. Synthesis and Structure

**Scheme 1:**
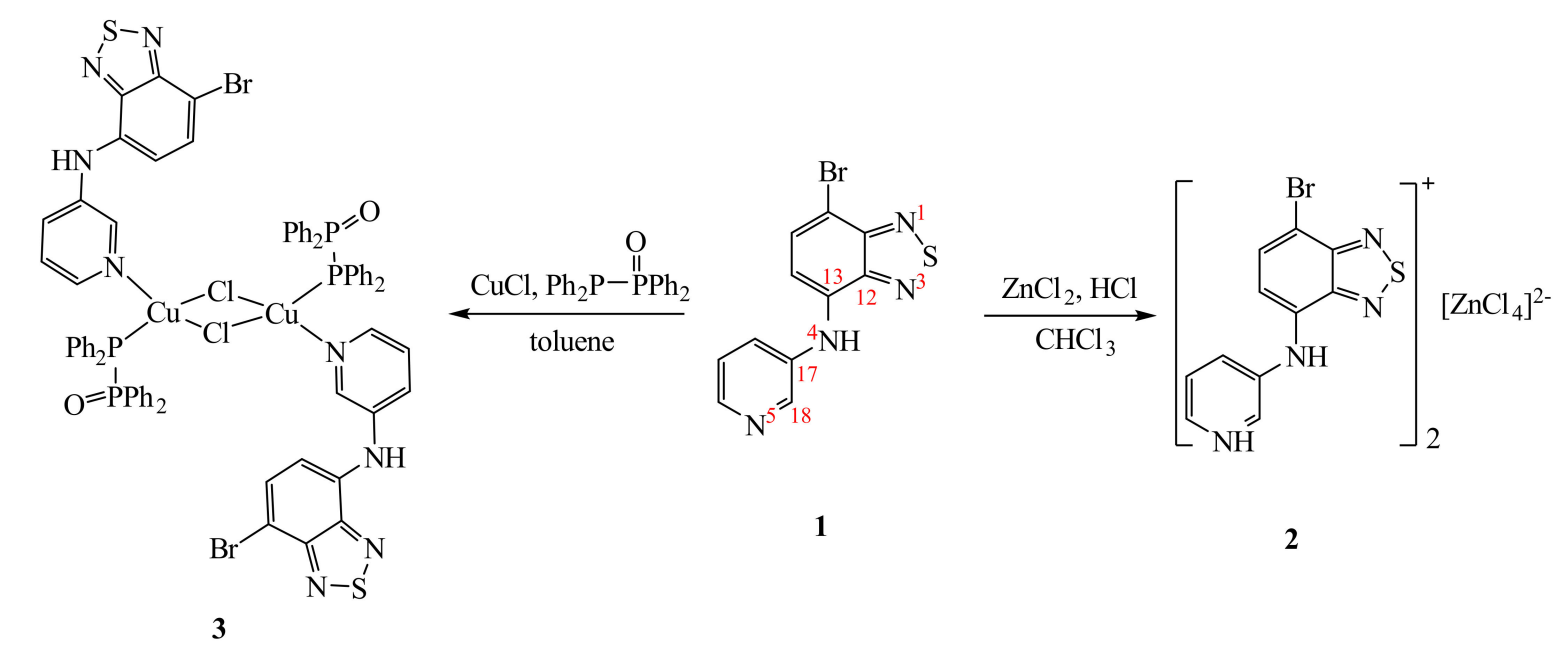
Synthesis of compounds **2** and **3∙**2C_7_H_8._

**Figure 1:**
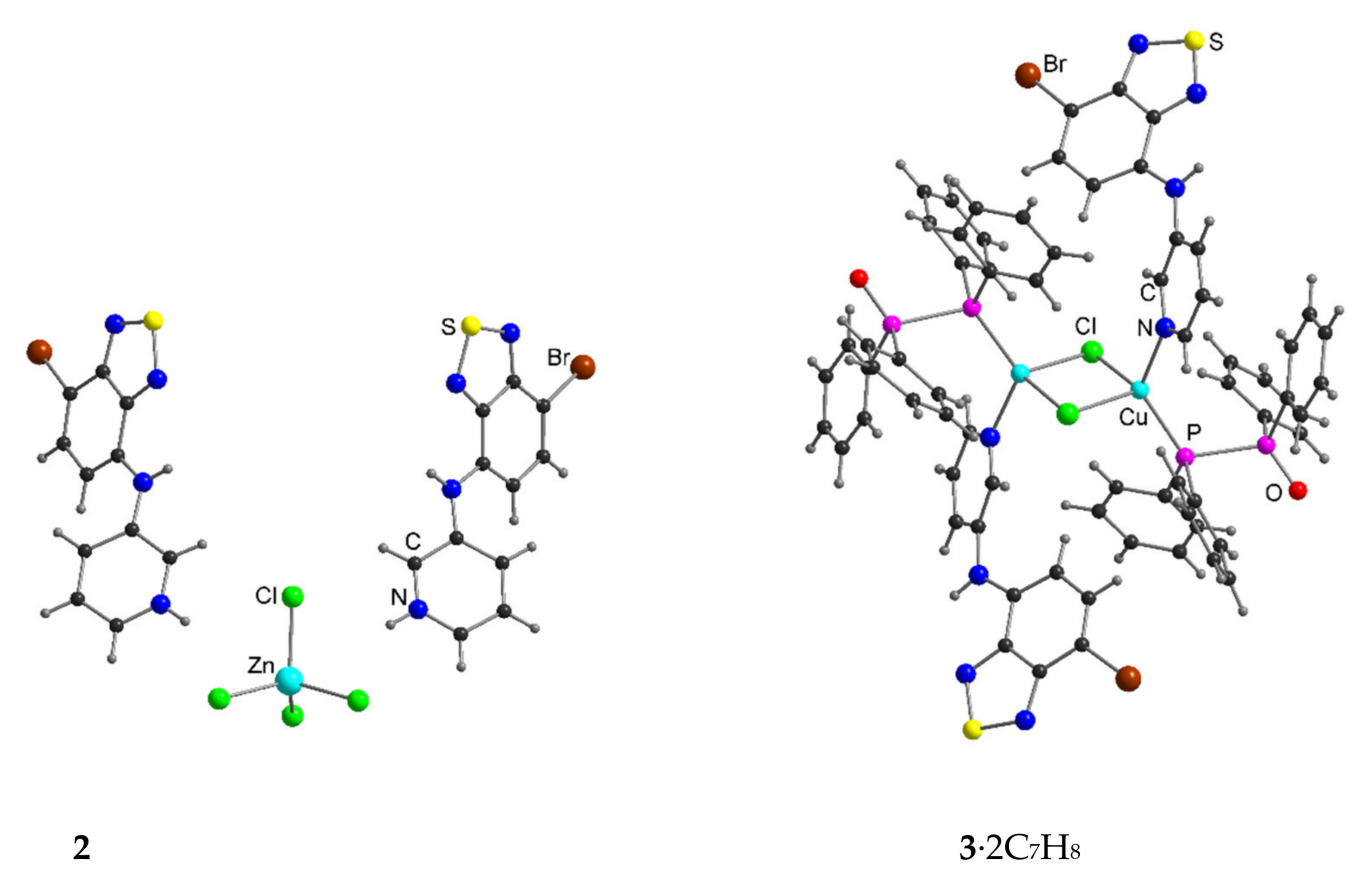
Structure of compounds **2** and **3**∙2C_7_H_8._

The reaction between compound **1** and ZnCl_2_ in chloroform results in the salt (*PyH-btd*)_2_(ZnCl_4_) (**2**), in which the benzothiadiazole *Py-btd* presents in the protonated form *PyH-btd*^+^ (Scheme 1, Figure 1). In this reaction, protonation of *Py-btd* at the nitrogen atom of the pyridyl group occurs due to the hydrochloric acid present in the solvent. Crystals suitable for XRD analysis were obtained by slow evaporation of a solution in methylene chloride. The target compound **2** in 60% yield was prepared by adding hydrochloric acid to the reaction mixture in chloroform. Protonated *PyH-btd*^+^ in the compound is not coordinated to zinc atom; the latter is surrounded by four chlorides and acts as a counterion. Despite the amino-benzothiadiazole moiety containing three donor N atoms, they do not coordinate the ZnCl_3_^–^ fragment (formed from ZnCl_2_ and one equivalent of HCl), and a zwitterionic complex [Zn(PyH-btd)Cl_3_] does not form for steric and/or electronic reasons. N^1^ and N^3^ atoms of the thiadiazole (for atoms numbering, see Scheme 1) have an interfering steric effect on the Br and (N)H atoms, respectively. The N^4^ atom of the amino group probably lacks donor strength to coordinate with the Zn^2+^ ion. The possible chelate coordination of the amino-benzothiadiazole moiety via N^3^ and N^4^ atoms is unfavorable, as discussed earlier for archetype 4-amino-2,1,3-benzothiadiazole [34]. Thus, in the case of the N^5^ site occupied by H^+^, the ZnCl_3_^–^ fragment prefers to attach one more Cl^–^ from HCl to form ZnCl_4_^2–^ rather than coordinate to *PyH-btd*^+^, although such complexes with positively charged N-donor ligands are quite common [35,36].

The reaction between **1**, tetraphenyldiphosphine monoxide (*PPO*), and copper(I) chloride in toluene gave the heteroligand coordination compound [Cu_2_Cl_2_(*Py-btd*)_2_{*PPO*}_2_]·2C_7_H_8_ (**3∙**2C_7_H_8_) (Scheme 1). Crystals suitable for XRD analysis were obtained by extraction of the substance with toluene in a two-section ampoule. The compound comprises binuclear complex **3**, in which Cu atoms are bound by bridging Cl atoms. Two *Py-btd* ligands are coordinated via the N^5^ atoms of the pyridyl unit, while the *PPO* ligand is coordinated via the trivalent phosphorus atom.

Powder diffraction patterns for samples of **2** and **3∙**2C_7_H_8_ (Figures S1 and S2) agree well with those simulated from single crystal XRD data, indicating the phase purity of the compounds.

All bond lengths in compounds **2** and **3**∙2C_7_H_8_ lie in the expected range, while the bonds of the *Py-btd* unit are close to those in compound **1 [19]**. Torsion angles C^12^–C^13^–N^4^–C^17^ for **1**-**3** are close to 180° (Table 1) owing to the conjugation of the amino group with the aromatic system of *btd*. The geometry of the *Py-btd* unit is similar in **1**,**2** and differs from that in **3**∙2C_7_H_8_ mainly by the torsion C^13^–N^4^–C^17^–C^18^ angle; however, the tilt of the pyridyl group relative to the amino-benzothiadiazole is similar in all the structures up to a change of place of N^5^ and C^20^ atoms.

**Figure 2:**
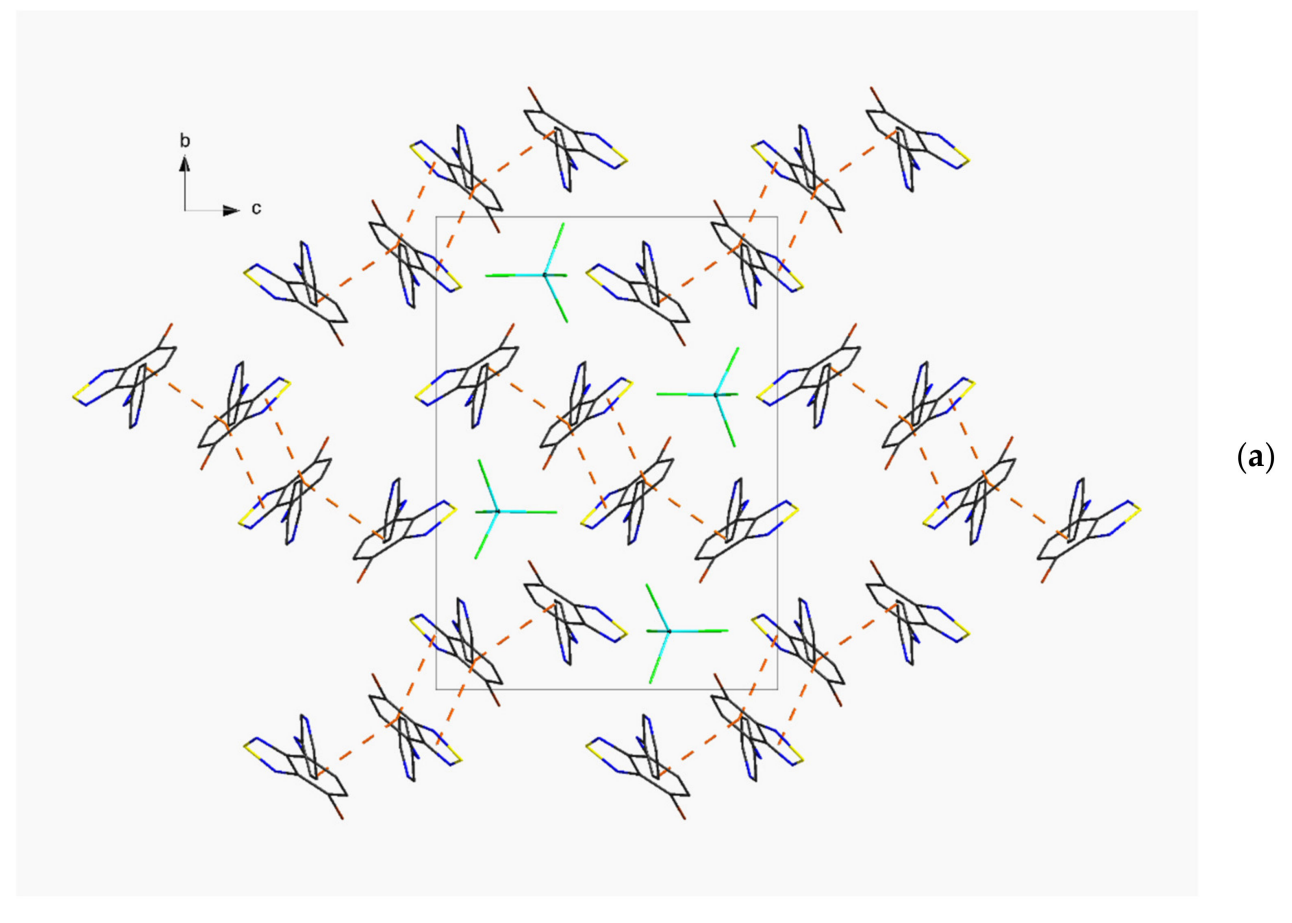

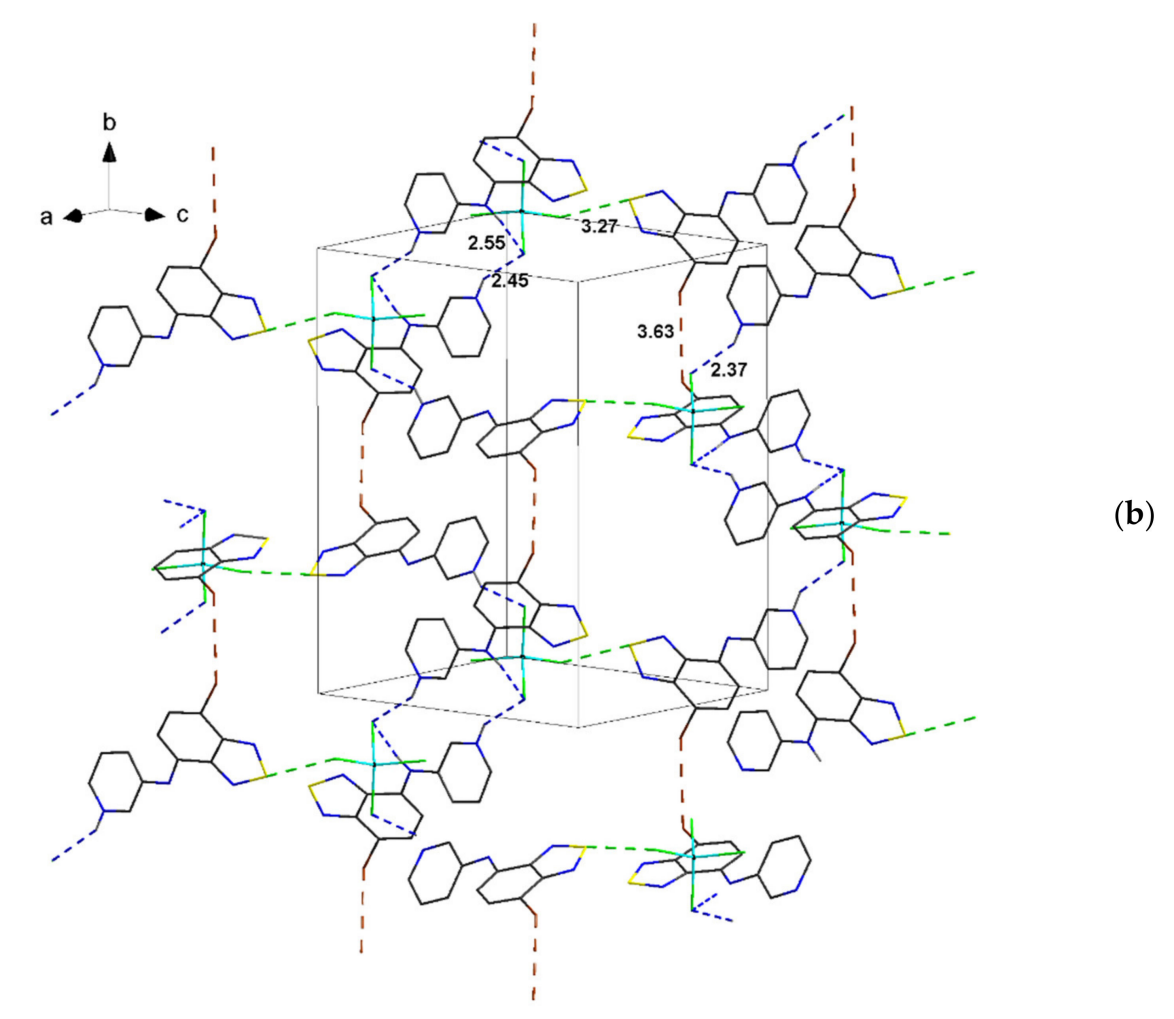
Crystal packing of **2** showing (**a**) π–π interactions (orange dashed lines) and (**b**) Br···Br (brown dashed lines), S···Cl contacts (green dashed lines) and N^4^H···Cl hydrogen bonds (blue dashed lines). Hydrogens except those participating in the hydrogen bonds are not shown.

**Figure 3:**
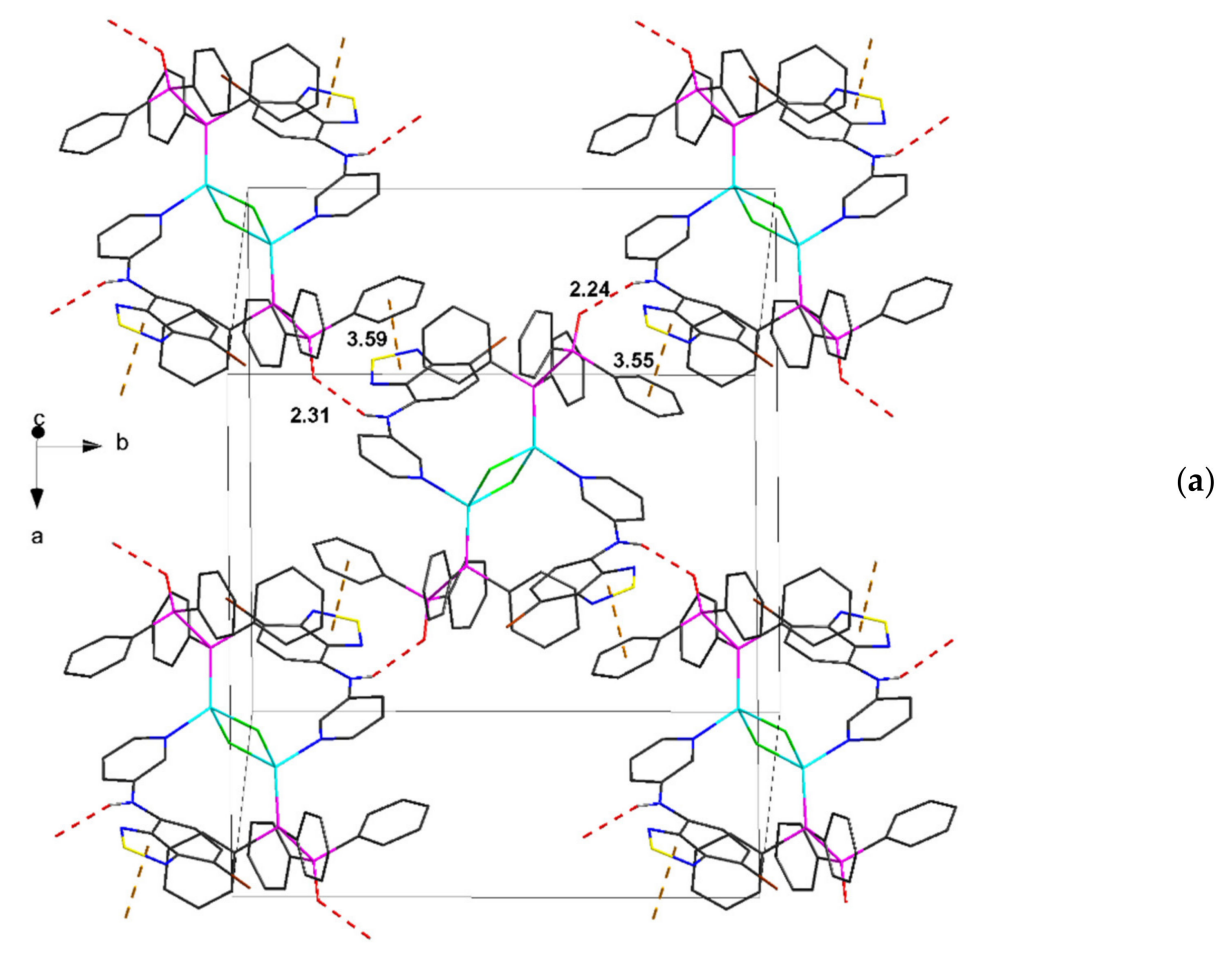

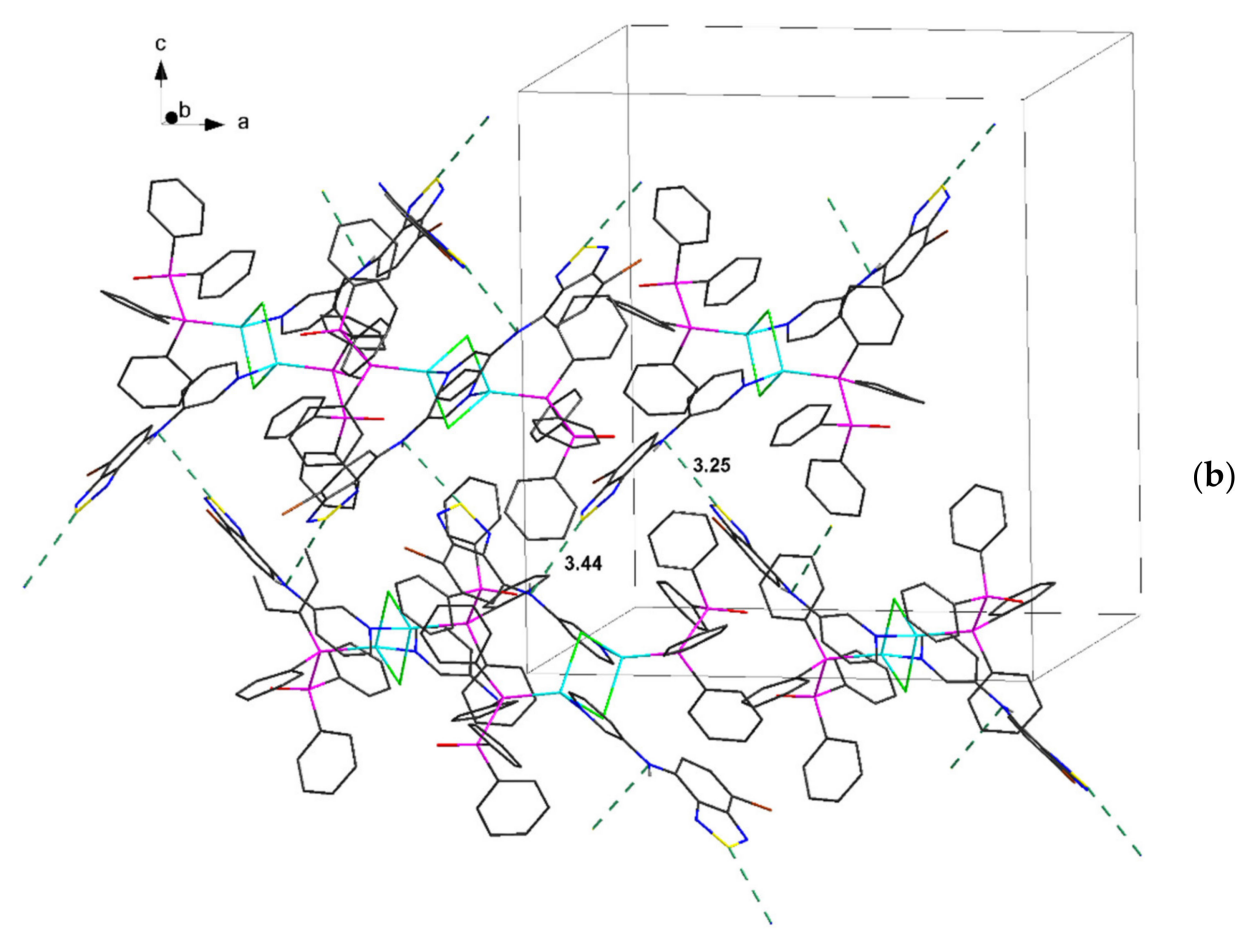
Crystal packing of **3**∙2C_7_H_8_ showing (**a**) π–π interactions (orange dashed lines) and N^4^H···O hydrogen bonds (red dashed lines) and (**b**) S···N^4^ contacts (green dashed lines). Toluene molecules and hydrogens except those participating in the hydrogen bonds are not shown.

In crystal **2**, *PyH-btd*^+^ cations are engaged in “head-to-tail” π–π stacking in tetramers, which, in turn, form a parquet pattern (Figure 2). In addition, the structure reveals intermolecular hydrogen bonds N^4^H···Cl and shortened contacts S···Cl and Br···Br. The structure **3**∙2C_7_H_8_ reveals π–π interaction between the *btd* unit and one of phenyl groups, intermolecular hydrogen bonds N^4^H···O and shortened contacts S···N (Figure 3). In **3**∙2C_7_H_8_, no specific interactions with Br atoms were found.

### 3.2. Photophysical Properties

**Figure 4:**
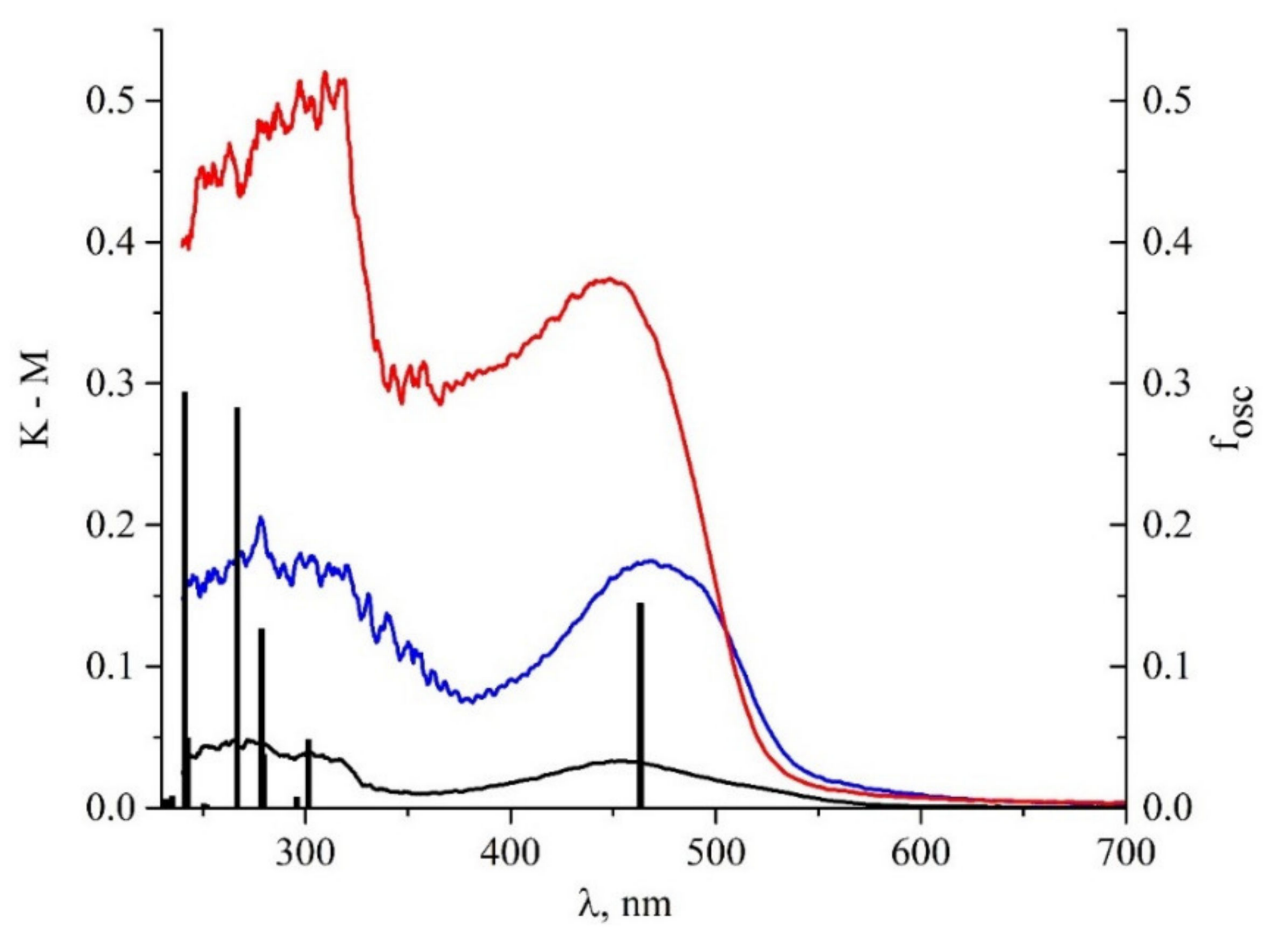
UV-Vis spectra of the solid samples of **1** (black) [19], **2** (red), and **3**∙2C_7_H_8_ (blue) mixed with BaSO_4_, presented in the form of Kubelka–Munk (K–M) functions; the intensities are recalculated to 0.005 mole fraction of the compounds. Black vertical lines represent calculated TD-DFT transitions (ZORA-PBE0/def2-TZVP(-f)) for **1**.

**Figure 5:**
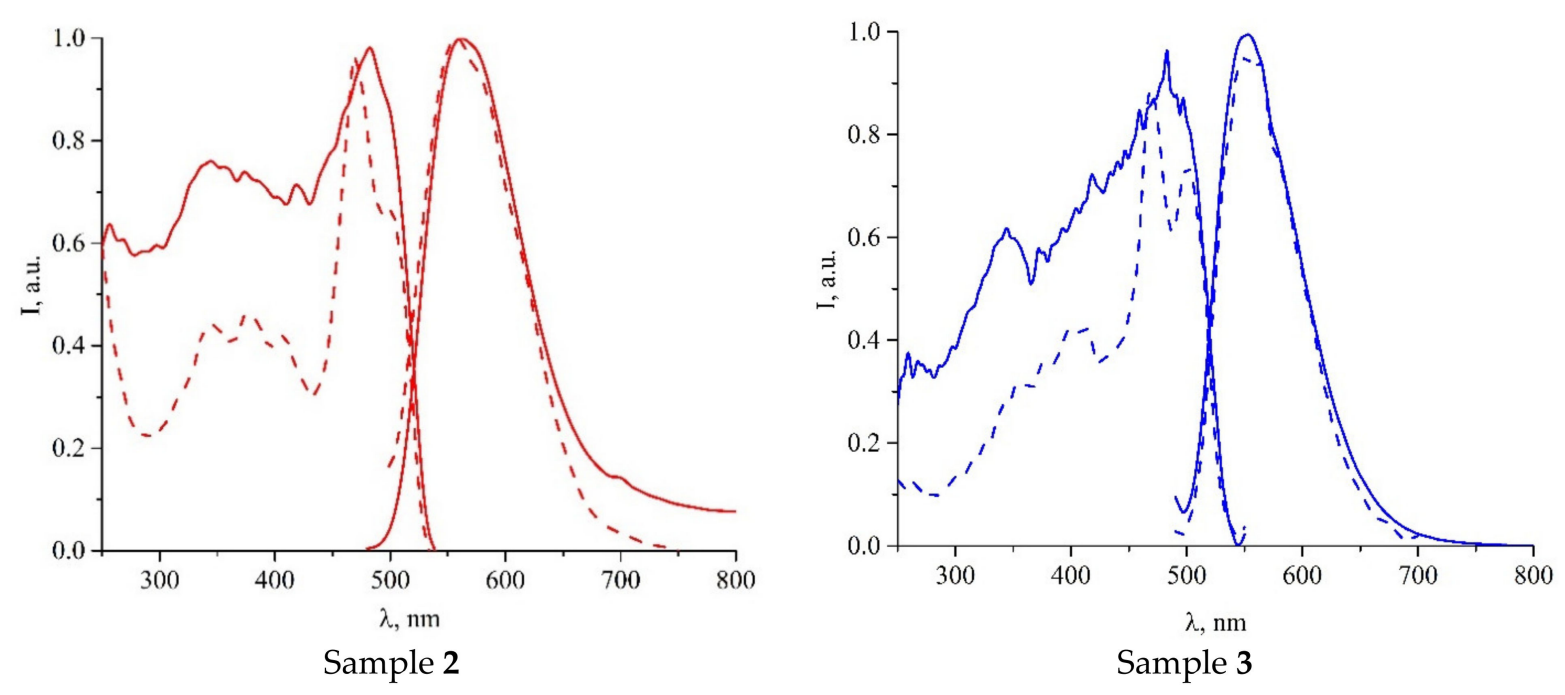
Normalized steady-state (solid lines) and delayed (100 μs) excitation and emission spectra of solid samples **2**,**3**.

For compounds **2** and **3**∙2C_7_H_8_ in the solid state (polycrystalline samples for luminescence studies and a mixture with BaSO_4_ for UV-vis), the UV-vis (Figure 4) and photoluminescence (Figure 5) spectra were measured. Compared to compound **1 [19]**, the shape of UV-vis spectra for **2** and **3**∙2C_7_H_8_ differs slightly (Table 2). They are characterized by a broad high-energy band at ca. 260–320 nm and a low-energy band at ca. 450 nm.

**Figure 6:**
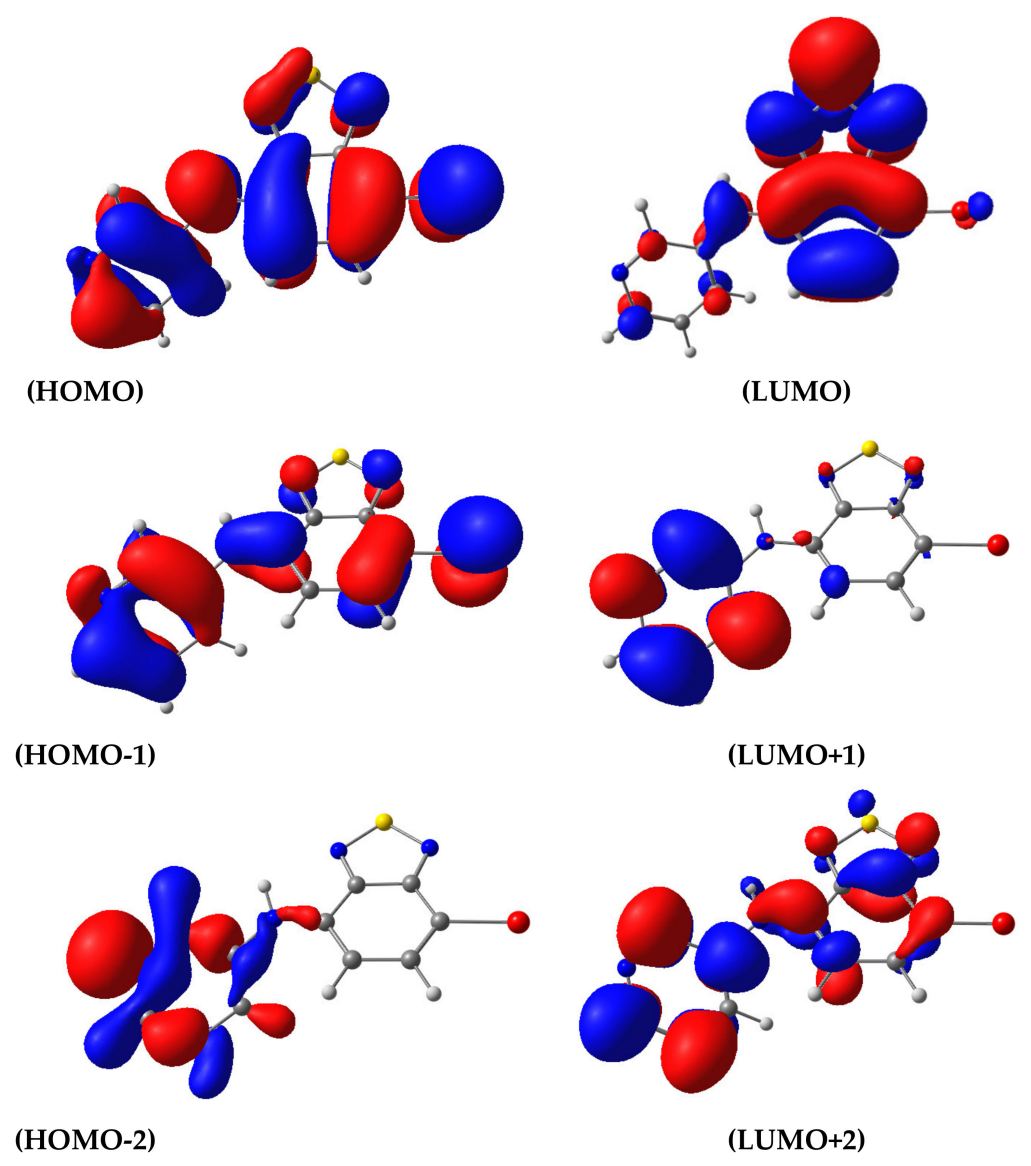
Frontier orbitals of **1** based on ZORA-PBE0/def2-TZVP(-f) calculation (isovalue = 0.02 a.u.).

According to the TD-DFT calculation on the ZORA-PBE0/def2-TZVP(-f) theory level (Table 3, Figure 6), the lowest energy absorption band of **1** at 455 nm corresponds mainly to a transition between the HOMO and LUMO, with intermediate character between charge transfer from pyridyl and Br to *btd* fragments and local excitation (Figure 6), as indicated by charge transfer (CT) number (Table 3). However, such transitions in related compounds are often referred to as charge transfer [37,38,39]. Multiple bands at ca. 270–300 correspond to a combination of promotions from HOMO, HOMO–1, and HOMO–2 to LUMO, LUMO+1 and LUMO+2.

Upon UV- or blue light irradiation, the compounds emit light in the visible region. Emission maximum hypsochromically shifts by 35 and 48 nm on going from **1** to **2** and to **3**∙2C_7_H_8_, respectively. Unexpectedly, compound **1** exhibits a microsecond order of luminescence lifetime at room temperature, which is not typical for purely organic substances [40,41] and indicates a triplet origin of the excited state. Delayed luminescence spectra of all compounds overlap well with the steady-state spectra (Figure 5), indicating that the transition occurs from one emitting species. The relatively long lifetime, similar for **1**–**3**, apparently originates from the heavy atom effect. The presence of a heavy atom, e.g., bromine, in the compounds promotes the intersystem crossing (ISC) process that occurs between the excited singlet and the triplet excited state [42,43]. To the best of our knowledge, only two works have been reported on purely organic phosphorescent Br-substituted 2,1,3-benzothiadiazoles [44,45], and a few more works have been devoted to organic non-bromine 2,1,3-benzothiadiazoles showing microsecond lifetimes [46,47,48]. Metal-containing phosphorescent benzothiadiazoles are more abundant, but examples of metals are predominantly represented by precious Pt(II) [49,50,51], Au(III) [52], Au(I) [53], Ir(III) [54,55] and Ru(II) [56].

## 4. Conclusions

To sum up, we modulated optical features of 4-bromo-7-(3-pyridylamino)-2,1,3-benzothiadiazole (*Py-btd*, **1**) by introducing Lewis acids, *viz* H^+^ and Cu^+^. The corresponding novel compounds (*PyH-btd*)_2_(ZnCl_4_) (**2**) and [Cu_2_Cl_2_(*Py-btd*)_2_{*PPO*}_2_]·2C_7_H_8_ (**3**·2C_7_H_8_; *PPO* = tetraphenyldiphosphine monoxide) were characterized by single crystal X-ray diffraction analysis and other physical–chemical methods. Unexpectedly, free *Py-btd* exhibits a microsecond order of luminescence lifetime at room temperature, which is not typical for purely organic substances and indicates a triplet origin of the excited state. The efficient singlet-to-triplet intersystem crossing, essential for a long-lifetime emission, is governed by the heavy atom effect. In our case, the heavy atom is bromine. Both Lewis adducts **2** and **3**·2C_7_H_8_ also possess a microsecond lifetime. As compared to free *Py-btd*, they reveal a noticeable hypsochromic shift of the emission band (with the energy difference of ca. 1000 and 1450 cm^−1^ for **2** and **3**·2C_7_H_8_, respectively), associated with the electronic influence of the Lewis acids. This motivates further design of phosphorescent benzothiadiazoles and their Lewis acid adducts.

## Figures and Tables

**Table 1:** Dihedral angles (deg.) and shortened intermolecular contacts (Å) for the 4-bromo-7-(3-pyridylamino)-2,1,3-benzothiadiazole (*Py-btd*) unit in compounds **1 [19]**, **2** and **3**∙2C_7_H_8_. For atom numbering, see Scheme 1.

Compound	C^12^–C^13^–N^4^–C^17^	C^13^–N^4^–C^17^–C^18^	S···*A* *	C_6_ Centroid–C_2_N_2_S, Centroid
**1**	180.0	149.4	2.99	3.58
**2**	158.1, 163.0	153.4, 155.3	3.27	3.57
**3**∙2C_7_H_8_	168.6, 174.0	30.9, 36.5	3.25, 3.43	3.55, 3.59 *

* For **1** and **3**∙2C_7_H_8_, *A* = N; for **2**, *A* = Cl. For **3**∙2C_7_H_8_, C_6_ moiety belongs to the phenyl group of the tetraphenyldiphosphine monoxide (*PPO*) ligand.

**Table 2:** Photophysical properties of compounds **1** [19], **2** and **3**∙2C_7_H_8._

Compound	λ_Em_, nm	λ_Em_, nm	τ, μs
**1**	265–320 (br), 455	600	9
**2**	255–315 (br), 445	565	18
**3**∙2C_7_H_8_	278–320 (br), 470	552	9

**Table 3:** Main transitions in *Py*-*btd* molecule. f–oscillator strength, H—HOMO, L—LUMO, CT—charge transfer number *.

State	λ, nm	f	Transition	Contribution	CT
1	463.2	0.1446	H→L	0.9869	0.503
2	301.4	0.0481	H→L+1	0.9400	0.700
3	295.7	0.0075	H-1→L	0.9555	0.728
4	279.5	0.0379	H-2→L H→L+2	0.6953 0.1774	0.838
5	278.5	0.1268	H-1→L	0.95548	0.385
6	269.2	0.0002	H-2→L H→L+2	0.6953 0.1774	0.573
7	266.7	0.2827	H-3→L H→L+2 H→L+3	0.178346 0.497742 0.249064	0.564

* CT calculated by Theodore 2.2 program [26]. For charge transfer process, the CT number is close to 1.0, for local excitations it is close to 0.

## Data Availability

Not applicable.

## Supplementary Materials

The following are available online. Table S1: Crystal data and struc-ture refinement for **2**–**3**. Figures S1 and S2: experimental and simulated powder diffraction pat-terns for **2**–**3**.

## Sample Availability

Samples of compounds **1**–**3** are available from the authors.
