# Supplementary material for: Unexpectedly Long Lifetime of the Excited State of Benzothiadiazole Derivative and Its Adducts with Lewis Acids
Source: Molecules. 2021 Apr 2;26(7):2030. doi: 10.3390/molecules26072030 (PMC8038179; doi:10.3390/molecules26072030)

checkCIF/PLATON report

Structure factors have been supplied for datablock(s) rk47

THIS REPORT IS FOR GUIDANCE ONLY. IF USED AS PART OF A REVIEW PROCEDURE FOR PUBLICATION, IT SHOULD NOT REPLACE THE EXPERTISE OF AN EXPERIENCED CRYSTALLOGRAPHIC REFEREE.

No syntax errors found. CIF dictionary Interpreting this report

Datablock: rk47

```
Bond precision:  C-C = 0.0091 A          Wavelength=0.71073

Cell:            a=11.5203(9)    b=18.2029(16)     c=13.1842(12)
                 alpha=90        beta=93.957(3)    gamma=90
Temperature:     150 K

                Calculated                  Reported
Volume          2758.2(4)                   2758.2(4)
Space group     P 21/c                      P 1 21/c 1
Hall group      -P 2ybc                     -P 2ybc
Moiety formula  2(C11 H8 Br N4 S), Cl4 Zn  2(C11 H8 Br N4 S), Cl4 Zn
Sum formula     C22 H16 Br2 Cl4 N8 S2 Zn   C22 H16 Br2 Cl4 N8 S2 Zn
Mr              823.54                      823.54
Dx,g cm-3       1.983                       1.983
Z               4                           4
Mu (mm-1)       4.359                       4.359
F000            1616.0                      1616.0
F000'           1618.58
h,k,lmax        13,21,15                    13,21,15
Nref            4560                        4555
Tmin,Tmax       0.599,0.804                 0.575,0.745
Tmin'           0.515

Correction method= # Reported T Limits: Tmin=0.575 Tmax=0.745
AbsCorr = MULTI-SCAN

Data completeness= 0.999          Theta(max)= 24.437

R(reflections)= 0.0478( 3230)     wR2(reflections)= 0.1176( 4555)

S = 1.014                  Npar= 358
```

The following ALERTS were generated. Each ALERT has the format
test-name_ALERT_alert-type_alert-level.
Click on the hyperlinks for more details of the test.

Alert level C

```
THETM01_ALERT_3_C  The value of sine(theta_max)/wavelength is less than 0.590
            Calculated sin(theta_max)/wavelength =    0.5821
PLAT213_ALERT_2_C Atom C211            has ADP max/min Ratio .....        3.6 oblate
PLAT213_ALERT_2_C Atom C217            has ADP max/min Ratio .....        3.6 oblate
PLAT213_ALERT_2_C Atom C219            has ADP max/min Ratio .....        3.4 prolat
PLAT341_ALERT_3_C Low Bond Precision on  C-C Bonds ...............     0.0091 Ang.
PLAT431_ALERT_2_C Short Inter HL..A Contact  Cl2      ..S22      .       3.27 Ang.
                                                1-x,1-y,1-z  =      3_666 Check
PLAT911_ALERT_3_C Missing FCF Refl Between Thmin & STh/L=    0.582          6 Report
```

Alert level G

```
PLAT002_ALERT_2_G Number of Distance or Angle Restraints on AtSite          4 Note
PLAT003_ALERT_2_G Number of Uiso or Uij Restrained non-H Atoms ...          1 Report
PLAT007_ALERT_5_G Number of Unrefined Donor-H Atoms ..............          2 Report
PLAT083_ALERT_2_G SHELXL Second Parameter in WGHT  Unusually Large       5.52 Why ?
PLAT172_ALERT_4_G The CIF-Embedded .res File Contains DFIX Records          1 Report
PLAT186_ALERT_4_G The CIF-Embedded .res File Contains ISOR Records          1 Report
PLAT794_ALERT_5_G Tentative Bond Valency for Zn1       (II)      .       1.96 Info
PLAT860_ALERT_3_G Number of Least-Squares Restraints .............          8 Note
PLAT909_ALERT_3_G Percentage of I>2sig(I) Data at Theta(Max) Still        49% Note
PLAT978_ALERT_2_G Number C-C Bonds with Positive Residual Density.          0 Info
```

---

0 **ALERT level A** = Most likely a serious problem - resolve or explain
0 **ALERT level B** = A potentially serious problem, consider carefully
7 **ALERT level C** = Check. Ensure it is not caused by an omission or oversight
10 **ALERT level G** = General information/check it is not something unexpected

0 ALERT type 1 CIF construction/syntax error, inconsistent or missing data
8 ALERT type 2 Indicator that the structure model may be wrong or deficient
5 ALERT type 3 Indicator that the structure quality may be low
2 ALERT type 4 Improvement, methodology, query or suggestion
2 ALERT type 5 Informative message, check

It is advisable to attempt to resolve as many as possible of the alerts in all categories. Often the minor alerts point to easily fixed oversights, errors and omissions in your CIF or refinement strategy, so attention to these fine details can be worthwhile. In order to resolve some of the more serious problems it may be necessary to carry out additional measurements or structure refinements. However, the purpose of your study may justify the reported deviations and the more serious of these should normally be commented upon in the discussion or experimental section of a paper or in the "special_details" fields of the CIF. checkCIF was carefully designed to identify outliers and unusual parameters, but every test has its limitations and alerts that are not important in a particular case may appear. Conversely, the absence of alerts does not guarantee there are no aspects of the results needing attention. It is up to the individual to critically assess their own results and, if necessary, seek expert advice.

Publication of your CIF in IUCr journals

A basic structural check has been run on your CIF. These basic checks will be run on all CIFs submitted for publication in IUCr journals (*Acta Crystallographica*, *Journal of Applied Crystallography*, *Journal of Synchrotron Radiation*); however, if you intend to submit to *Acta Crystallographica Section C* or *E* or *IUCrData*, you should make sure that full publication checks are run on the final version of your CIF prior to submission.

Publication of your CIF in other journals

Please refer to the *Notes for Authors* of the relevant journal for any special instructions relating to CIF submission.

PLATON version of 05/12/2020; check.def file version of 05/12/2020

Datablock rk47 - ellipsoid plot

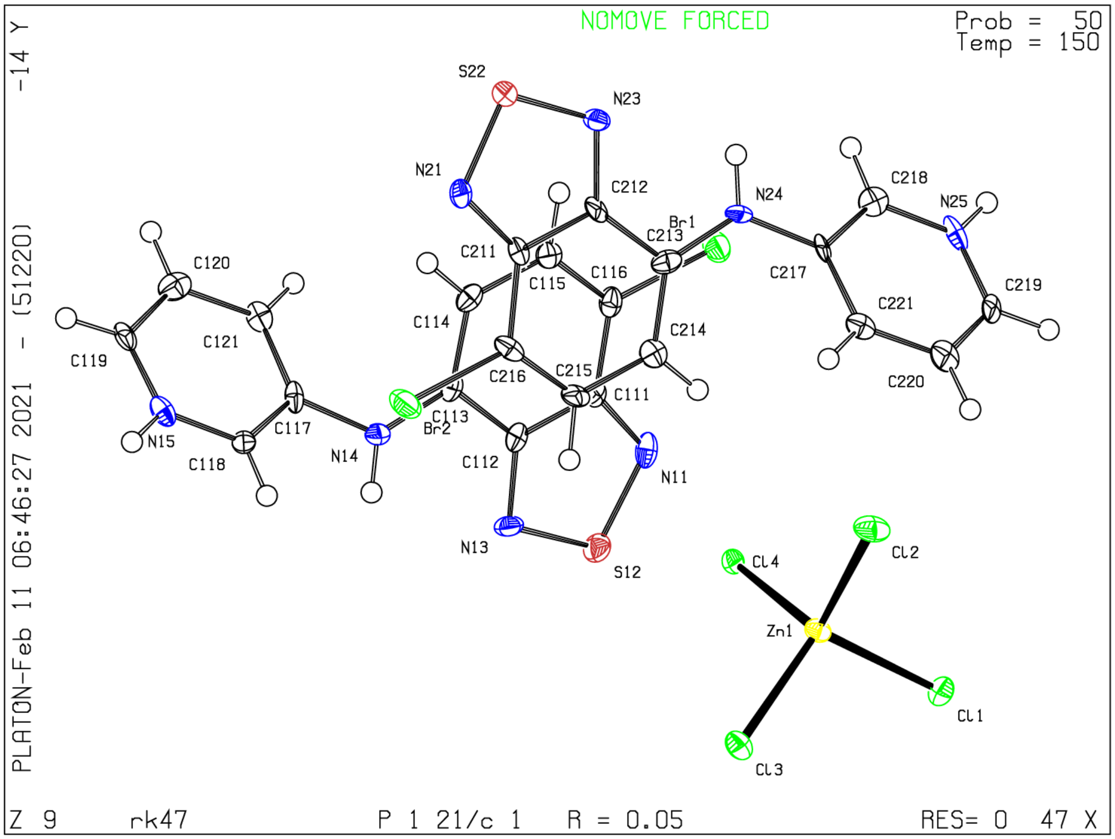

Supplement: Supplementary file 1 [file molecules-26-02030-s001.zip › rk47_cifreport.pdf]
