# Supplementary material for: Unexpectedly Long Lifetime of the Excited State of Benzothiadiazole Derivative and Its Adducts with Lewis Acids
Source: Molecules. 2021 Apr 2;26(7):2030. doi: 10.3390/molecules26072030 (PMC8038179; doi:10.3390/molecules26072030)

checkCIF/PLATON report

Structure factors have been supplied for datablock(s) rk61

THIS REPORT IS FOR GUIDANCE ONLY. IF USED AS PART OF A REVIEW PROCEDURE FOR PUBLICATION, IT SHOULD NOT REPLACE THE EXPERTISE OF AN EXPERIENCED CRYSTALLOGRAPHIC REFEREE.

No syntax errors found. CIF dictionary Interpreting this report

Datablock: rk61

Bond precision: C-C = 0.0237 A Wavelength=0.71073

Cell:	a=19.7043(12)	b=18.9722(12)	c=22.2962(17)
	alpha=90	beta=90	gamma=90
Temperature:	150 K		

	Calculated	Reported
Volume	8335.1(10)	8335.1(10)
Space group	P n a 21	P n a 21
Hall group	P 2c -2n	P 2c -2n
Moiety formula	C70 H54 Br2 Cl2 Cu2 N8 O2 P4 S2, 3(C7 H8)	C70 H54 Br2 Cl2 Cu2 N8 O2 P4 S2, 3(C7 H8)
Sum formula	C91 H78 Br2 Cl2 Cu2 N8 O2 P4 S2	C91 H78 Br2 Cl2 Cu2 N8 O2 P4 S2
Mr	1861.42	1861.41
Dx,g cm-3	1.483	1.483
Z	4	4
Mu (mm-1)	1.715	1.715
F000	3800.0	3800.0
F000’	3805.04	
h,k,lmax	22,22,25	0,0,0
Nref	13748[7072]	11798
Tmin,Tmax	0.742,0.787	0.643,0.745
Tmin’	0.667	

Correction method= # Reported T Limits: Tmin=0.643 Tmax=0.745
AbsCorr = MULTI-SCAN

Data completeness= 1.67/0.86 Theta(max)= 24.438

R(reflections)= 0.0716(6228) wR2(reflections)= 0.1520(11798)

S = 0.910 Npar= 883

```
The following ALERTS were generated. Each ALERT has the format
       test-name_ALERT_alert-type_alert-level.
Click on the hyperlinks for more details of the test.
```

Alert level B

```
PLAT341_ALERT_3_B Low Bond Precision on  C-C Bonds ...............    0.02374 Ang.
```

Alert level C

```
STRVA01_ALERT_4_C           Flack test results are ambiguous.
           From the CIF: _refine_ls_abs_structure_Flack    0.430
           From the CIF: _refine_ls_abs_structure_Flack_su    0.020
THETM01_ALERT_3_C  The value of sine(theta_max)/wavelength is less than 0.590
            Calculated sin(theta_max)/wavelength =    0.5821
PLAT090_ALERT_3_C Poor Data / Parameter Ratio (Zmax > 18) ........       7.98 Note
PLAT202_ALERT_3_C Isotropic non-H Atoms in Anion/Solvent .........         21 Check
              C31S    C32S    C33S    C34S    C35S    C36S               etc.
PLAT213_ALERT_2_C Atom C13             has ADP max/min Ratio .....        3.1 prolat
PLAT213_ALERT_2_C Atom C51             has ADP max/min Ratio .....        3.2 oblate
PLAT213_ALERT_2_C Atom C53             has ADP max/min Ratio .....        3.1 prolat
PLAT213_ALERT_2_C Atom C71             has ADP max/min Ratio .....        3.8 prolat
PLAT220_ALERT_2_C NonSolvent   Resd 1  C   Ueq(max)/Ueq(min) Range        4.1 Ratio
PLAT234_ALERT_4_C Large Hirshfeld Difference S12      --N13      .       0.17 Ang.
PLAT241_ALERT_2_C High   'MainMol' Ueq as Compared to Neighbors of        C55 Check
PLAT242_ALERT_2_C Low    'MainMol' Ueq as Compared to Neighbors of        C61 Check
PLAT242_ALERT_2_C Low    'MainMol' Ueq as Compared to Neighbors of       C116 Check
PLAT243_ALERT_4_C High   'Solvent' Ueq as Compared to Neighbors of       C34S Check
PLAT243_ALERT_4_C High   'Solvent' Ueq as Compared to Neighbors of       C11S Check
PLAT243_ALERT_4_C High   'Solvent' Ueq as Compared to Neighbors of       C13S Check
PLAT243_ALERT_4_C High   'Solvent' Ueq as Compared to Neighbors of       C14S Check
PLAT243_ALERT_4_C High   'Solvent' Ueq as Compared to Neighbors of       C21S Check
PLAT243_ALERT_4_C High   'Solvent' Ueq as Compared to Neighbors of       C23S Check
PLAT243_ALERT_4_C High   'Solvent' Ueq as Compared to Neighbors of       C25S Check
PLAT244_ALERT_4_C Low    'Solvent' Ueq as Compared to Neighbors of       C31S Check
PLAT244_ALERT_4_C Low    'Solvent' Ueq as Compared to Neighbors of       C32S Check
PLAT244_ALERT_4_C Low    'Solvent' Ueq as Compared to Neighbors of       C35S Check
PLAT244_ALERT_4_C Low    'Solvent' Ueq as Compared to Neighbors of       C12S Check
PLAT244_ALERT_4_C Low    'Solvent' Ueq as Compared to Neighbors of       C16S Check
PLAT244_ALERT_4_C Low    'Solvent' Ueq as Compared to Neighbors of       C22S Check
PLAT244_ALERT_4_C Low    'Solvent' Ueq as Compared to Neighbors of       C26S Check
PLAT331_ALERT_2_C Small Aver Phenyl C-C Dist C21      --C26      .       1.36 Ang.
PLAT331_ALERT_2_C Small Aver Phenyl C-C Dist C31      --C36      .       1.37 Ang.
PLAT362_ALERT_2_C Short  C(sp3)-C(sp2) Bond  C21S     - C27S     .       1.37 Ang.
PLAT411_ALERT_2_C Short Inter H...H Contact  H22      ..H23S     .       2.08 Ang.
                                          1/2+x,3/2-y,z  =      3_565 Check
PLAT906_ALERT_3_C Large K Value in the Analysis of Variance ......      3.841 Check
PLAT911_ALERT_3_C Missing FCF Refl Between Thmin & STh/L=    0.582         26 Report
```

Alert level G

```
PLAT002_ALERT_2_G Number of Distance or Angle Restraints on AtSite         17 Note
PLAT003_ALERT_2_G Number of Uiso or Uij Restrained non-H Atoms ...         75 Report
PLAT007_ALERT_5_G Number of Unrefined Donor-H Atoms ..............          2 Report
PLAT172_ALERT_4_G The CIF-Embedded .res File Contains DFIX Records          2 Report
PLAT174_ALERT_4_G The CIF-Embedded .res File Contains FLAT Records          2 Report
PLAT176_ALERT_4_G The CIF-Embedded .res File Contains SADI Records          4 Report
PLAT186_ALERT_4_G The CIF-Embedded .res File Contains ISOR Records          4 Report
PLAT187_ALERT_4_G The CIF-Embedded .res File Contains RIGU Records         12 Report
PLAT232_ALERT_2_G Hirshfeld Test Diff (M-X)  Cu1      --Cl2      .        7.0 s.u.
PLAT302_ALERT_4_G Anion/Solvent/Minor-Residue Disorder (Resd  2  )        14% Note
```

```
PLAT432_ALERT_2_G Short Inter X...Y Contact  C16S     ..C38S            3.07 Ang.
                                         3/2-x,-1/2+y,1/2+z  =      4_645 Check
PLAT860_ALERT_3_G Number of Least-Squares Restraints .............        612 Note
PLAT870_ALERT_4_G ALERTS Related to Twinning Effects Suppressed ..          ! Info
PLAT883_ALERT_1_G No Info/Value for _atom_sites_solution_primary .     Please Do !
PLAT910_ALERT_3_G Missing # of FCF Reflection(s) Below Theta(Min).          2 Note
PLAT933_ALERT_2_G Number of OMIT Records in Embedded .res File ...          5 Note
PLAT941_ALERT_3_G Average HKL Measurement Multiplicity ...........        1.7 Low
PLAT961_ALERT_5_G Dataset Contains no Negative Intensities .......     Please Check
```

```
 0 ALERT level A = Most likely a serious problem - resolve or explain
 1 ALERT level B = A potentially serious problem, consider carefully
33 ALERT level C = Check. Ensure it is not caused by an omission or oversight
18 ALERT level G = General information/check it is not something unexpected

 1 ALERT type 1 CIF construction/syntax error, inconsistent or missing data
17 ALERT type 2 Indicator that the structure model may be wrong or deficient
 9 ALERT type 3 Indicator that the structure quality may be low
23 ALERT type 4 Improvement, methodology, query or suggestion
 2 ALERT type 5 Informative message, check
```

It is advisable to attempt to resolve as many as possible of the alerts in all categories. Often the minor alerts point to easily fixed oversights, errors and omissions in your CIF or refinement strategy, so attention to these fine details can be worthwhile. In order to resolve some of the more serious problems it may be necessary to carry out additional measurements or structure refinements. However, the purpose of your study may justify the reported deviations and the more serious of these should normally be commented upon in the discussion or experimental section of a paper or in the "special_details" fields of the CIF. checkCIF was carefully designed to identify outliers and unusual parameters, but every test has its limitations and alerts that are not important in a particular case may appear. Conversely, the absence of alerts does not guarantee there are no aspects of the results needing attention. It is up to the individual to critically assess their own results and, if necessary, seek expert advice.

Publication of your CIF in IUCr journals

A basic structural check has been run on your CIF. These basic checks will be run on all CIFs submitted for publication in IUCr journals (*Acta Crystallographica*, *Journal of Applied Crystallography*, *Journal of Synchrotron Radiation*); however, if you intend to submit to *Acta Crystallographica Section C* or *E* or *IUCrData*, you should make sure that full publication checks are run on the final version of your CIF prior to submission.

Publication of your CIF in other journals

Please refer to the *Notes for Authors* of the relevant journal for any special instructions relating to CIF submission.

PLATON version of 05/12/2020; check.def file version of 05/12/2020

Datablock rk61 - ellipsoid plot

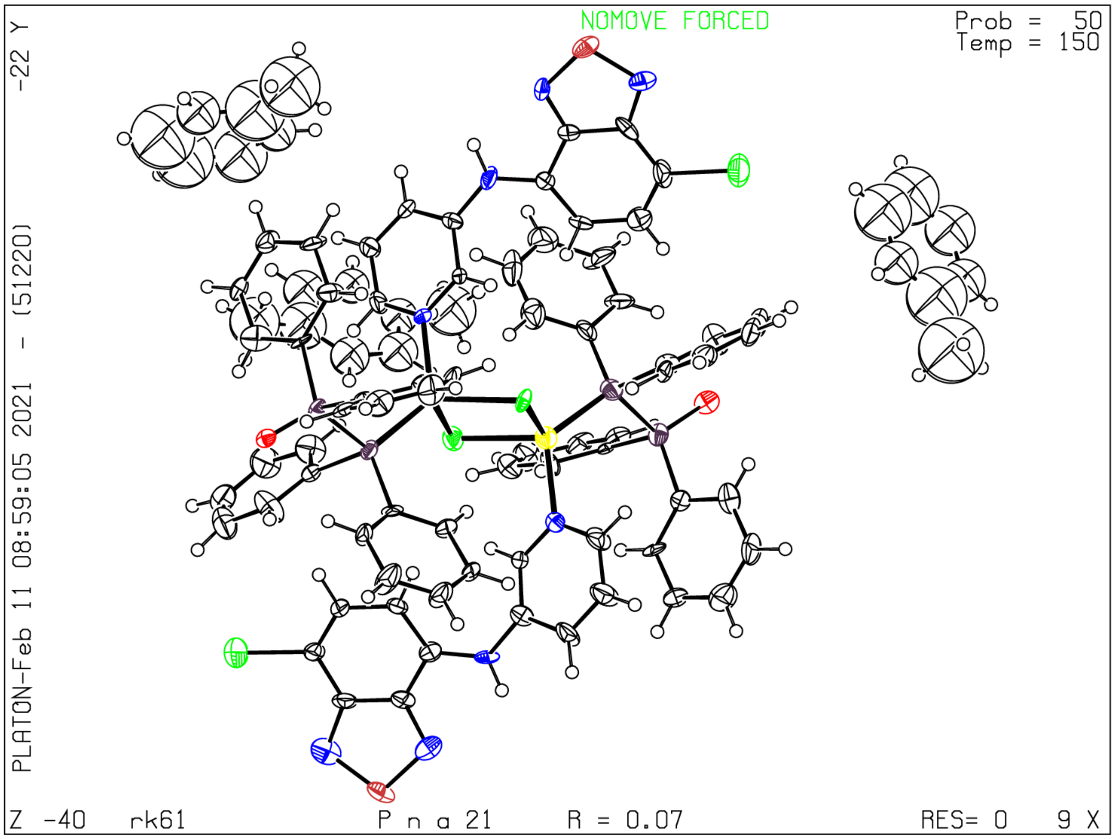

Supplement: Supplementary file 1 [file molecules-26-02030-s001.zip › RK61_checkcif.pdf]
