# Supplementary material for: Unexpectedly Long Lifetime of the Excited State of Benzothiadiazole Derivative and Its Adducts with Lewis Acids
Source: Molecules. 2021 Apr 2;26(7):2030. doi: 10.3390/molecules26072030 (PMC8038179; doi:10.3390/molecules26072030)
Supplement: Supplementary file 1 [file molecules-26-02030-s001.zip › SI-1160178/Supporting Information.pdf]

Supporting Information

Unexpectedly long lifetime of the excited state of benzothiadiazole derivative and its adducts with Lewis acids

by

Radmir M. Khisamov, Taisiya S. Sukhikh, Sergey N. Konchenko

Table S1. Crystal data and structure refinement for the compounds.

Identification code	**2**	**3**·$2C_7H_8$
Empirical formula	$C_{22}H_{16}Br_2Cl_4N_8S_2Zn$	$C_{91}H_{78}Br_2Cl_2Cu_2N_8O_2P_4S_2$
Formula weight	823.54	1861.41
Temperature/K	150(2)	150(2)
Crystal system	monoclinic	orthorhombic
Space group	$P2_1/c$	$Pna2_1$
a/Å	11.5203(9)	19.7043(12)
b/Å	18.2029(16)	18.9722(12)
c/Å	13.1842(12)	22.2962(17)
β/°	93.957(3)	90
Volume/Å^3	2758.2(4)	8335.1(10)
Z	4	4
ρ_{calc}g/cm^3	1.983	1.483
μ/mm^{-1}	4.359	1.715
F(000)	1616.0	3800.0
Crystal size/mm^3	0.15 × 0.1 × 0.05	0.23 × 0.15 × 0.14
Radiation	MoKα (λ = 0.71073)	MoKα (λ = 0.71073)
2Θ range for data collection/ ^{c}	3.544 to 48.874	3.496 to 48.876
Reflections collected	25850	11798
Independent reflections	4555 [R_{int} = 0.0908, R_{sigma} = 0.0790]	11798 [R_{int} = 0.089, R_{sigma} = 0.3417]
Data/restraints/parameters	4555/8/358	11798/612/883
Goodness-of-fit on F^2	1.014	0.910
Final R indexes [I>=2σ (I)]	R_1 = 0.0478, wR_2 = 0.1044	R_1 = 0.0716, wR_2 = 0.1441
Final R indexes [all data]	R_1 = 0.0833, wR_2 = 0.1176	R_1 = 0.1144, wR_2 = 0.1520
Largest diff. peak/hole / e Å^{-3}	1.45/-0.91	1.36/-0.76
Flack parameter		0.43(2)
