# Supplementary material for: Unexpectedly Long Lifetime of the Excited State of Benzothiadiazole Derivative and Its Adducts with Lewis Acids
Source: Molecules. 2021 Apr 2;26(7):2030. doi: 10.3390/molecules26072030 (PMC8038179; doi:10.3390/molecules26072030)
Supplement: Supplementary file 1 [file molecules-26-02030-s001.zip › Supporting Information.docx]

**Supporting Information**

**Unexpectedly long lifetime of the excited state of benzothiadiazole derivative and its adducts with Lewis acids**

**by**

**Radmir M. Khisamov, Taisiya S. Sukhikh, Sergey N. Konchenko**

**Table S1. Crystal data and structure refinement for the compounds.**

| Identification code | **2** | **3∙**2C_7_H_8_ |
| --- | --- | --- |
| Empirical formula | C_22_H_16_Br_2_Cl_4_N_8_S_2_Zn | C_91_H_78_Br_2_Cl_2_Cu_2_N_8_O_2_P_4_S_2_ |
| Formula weight | 823.54 | 1861.41 |
| Temperature/K | 150(2) | 150(2) |
| Crystal system | monoclinic | orthorhombic |
| Space group | *P*2_1_/*c* | *Pna*2_1_ |
| a/Å | 11.5203(9) | 19.7043(12) |
| b/Å | 18.2029(16) | 18.9722(12) |
| c/Å | 13.1842(12) | 22.2962(17) |
| β/° | 93.957(3) | 90 |
| Volume/Å^3^ | 2758.2(4) | 8335.1(10) |
| Z | 4 | 4 |
| ρ_calc_g/cm^3^ | 1.983 | 1.483 |
| μ/mm^‑1^ | 4.359 | 1.715 |
| F(000) | 1616.0 | 3800.0 |
| Crystal size/mm^3^ | 0.15 × 0.1 × 0.05 | 0.23 × 0.15 × 0.14 |
| Radiation | MoKα (λ = 0.71073) | MoKα (λ = 0.71073) |
| 2Θ range for data collection/° | 3.544 to 48.874 | 3.496 to 48.876 |
| Reflections collected | 25850 | 11798 |
| Independent reflections | 4555 [R_int_ = 0.0908, R_sigma_ = 0.0790] | 11798 [R_int_ = 0.089, R_sigma_ = 0.3417] |
| Data/restraints/parameters | 4555/8/358 | 11798/612/883 |
| Goodness-of-fit on F^2^ | 1.014 | 0.910 |
| Final R indexes [I>=2σ (I)] | R_1_ = 0.0478, wR_2_ = 0.1044 | R_1_ = 0.0716, wR_2_ = 0.1441 |
| Final R indexes [all data] | R_1_ = 0.0833, wR_2_ = 0.1176 | R_1_ = 0.1144, wR_2_ = 0.1520 |
| Largest diff. peak/hole / e Å^-3^ | 1.45/-0.91 | 1.36/-0.76 |
| Flack parameter |  | 0.43(2) |
